# Improved eradication of Clostridium difficile spores from toilets of hospitalized patients using an accelerated hydrogen peroxide as the cleaning agent

**DOI:** 10.1186/1471-2334-10-268

**Published:** 2010-09-15

**Authors:** Michelle J Alfa, Evelyn Lo, Alana Wald, Christine Dueck, Pat DeGagne, Godfrey KM Harding

**Affiliations:** 1 Department of Medical Microbiology, University of Manitoba, Winnipeg, Manitoba, Canada; 2 Diagnostic Services of Manitoba, Microbiology Discipline, St. Boniface General Hospital site, Winnipeg, Manitoba, Canada; 3 Microbiology Laboratory, St. Boniface Research, Winnipeg, Manitoba, Canada; 4 St. Boniface General Hospital, Infection Control, Winnipeg, Manitoba, Canada

## Abstract

**Background:**

*C. difficle *spores in the environment of patients with *C. difficile *associated disease (CDAD) are difficult to eliminate. Bleach (5000 ppm) has been advocated as an effective disinfectant for the environmental surfaces of patients with CDAD. Few alternatives to bleach for non-outbreak conditions have been evaluated in controlled healthcare studies.

**Methods:**

This study was a prospective clinical comparison during non-outbreak conditions of the efficacy of an accelerated hydrogen peroxide cleaner (0.5% AHP) to the currently used stabilized hydrogen peroxide cleaner (0.05% SHP at manufacturer recommended use-dilution) with respect to spore removal from toilets in a tertiary care facility. The toilets used by patients who had diarrhea with and without *C. difficile *associated disease (CDAD) were cultured for *C. difficile *and were monitored using an ultraviolet mark (UVM) to assess cleaning compliance on a daily basis 5 days per week. A total of 243 patients and 714 samples were analysed. The culture results were included in the analysis only if the UVM audit from the same day confirmed that the toilet had been cleaned.

**Results:**

Our data demonstrated that the efficacy of spore killing is formulation specific and cannot be generalized. The Oxivir_TB_^® ^AHP formulation resulted in statistically significantly (p = 0.0023) lower levels of toxigenic *C. difficile *spores in toilets of patients with CDAD compared to the SHP formulation that was routinely being used (28% vs 45% culture positive). The background level of toxigenic *C. difficile *spores was 10% in toilets of patients with diarrhea not due to CDAD. The UVM audit indicated that despite the enhanced twice-daily cleaning protocol for CDAD patients cleaning was not achieved on approximately 30 - 40% of the days tested.

**Conclusion:**

Our data indicate that the AHP formulation evaluated that has some sporicidal activity was significantly better than the currently used SHP formulation. This AHP formulation provides a one-step process that significantly lowers the *C. difficile *spore level in toilets during non-outbreak conditions without the workplace safety concerns associated with 5000 ppm bleach.

## Background

Toxigenic *Clostridium difficile *causes a significant number of enteric infections world-wide [1-5]. In Manitoba, where C. *difficile *associated diarrhea (CDAD) is reportable, there were 985 cases of CDAD compared to 512 cases of all other enteric bacterial infections combined in 2007 [6]. The incidence of CDAD ranges from 3.4 cases/1000 admissions up to 50 cases/1000 admissions [1,7]. The rates of CDAD per 100,000 population in the USA have almost doubled between 1996 and 2003 [8].

A major reservoir linked to nosocomial infections is thought to be the environment of healthcare facilities that are contaminated with the *C. difficile *spores shed by patients with CDAD [1,7,9,8,11]. The persistence of *C. difficile *spores has been well documented with toilets having the highest levels [1,9,12]. Reducing *C. difficile *spores from environmental sources is challenging as few surface disinfectant and/or cleaning agents have sporocidal activity in a short enough time-frame (e.g. 3 minutes) to be effective [13].

National guidelines in Canada do not recommend disinfectants for routine housekeeping [14] but many healthcare facilities use bleach at a 1:10 dilution (5,000 ppm) and increase cleaning from once to twice daily for patients with CDAD as per PIDAC[9,10,12,15]. Despite being widely accepted, neither Wilcox's original study [16] nor Eckstein's recent study [9] were able to demonstrate complete eradication of spores when 5,000 ppm bleach was used for cleaning/disinfecting toilet facilities of patients with CDAD. In most published studies, bleach at 5,000 ppm was combined with other heightened strategies such as improved housekeeping, enhanced compliance with infection control isolation precautions, and increased education [10]. Although 5,000 ppm chlorine bleach is an effective sporicidal agent, there are significant workplace safety concerns related to using bleach and it requires a two step process (i.e. must be wiped off using water). There are no published studies that have audited cleaning compliance in conjunction with evaluating bleach alternatives (that have some sporicidal activity) that could be used for environmental cleaning of CDAD patient toilets during non-outbreak conditions.

The objective of this research was to determine if the presence of *C. difficile *spores in toilets of patients with CDAD could be reduced in non-outbreak conditions when a non-bleach based disinfecting agent that had some sporicidal activity was used for cleaning toilets. To ensure that the intervention was used the toilets were audited with UVM and only if the toilet had received cleaning was the culture date included in the overall analysis.

## Methods

### 1. Microorganisms used in this study

*C. difficile *strain 726 was a clinical isolate that was used for spore preparation as described by Alfa et al [17]. The spore preparation of ~10^6 ^spores/mL was stored in 45% alcohol and the same batch of spores was used for all testing.

### 2. Preliminary testing to select the intervention formulation

The efficacy of various disinfectant solutions at killing spores of *C. difficile *was assessed using suspension and surface testing. The use of ATS (artificial test soil) as an organic challenge and the recovery of residual *C. difficile *spores from inoculated toilet seats by Rodac plates is described by Alfa et al [17]. Five different chemical disinfectants were evaluated; PerDiem at 1:16 use-dilution (research based use-dilution that is not recommended by the manufacturer), Oxivir_TB_^® ^used directly (manufacturer's recommended use-dilution), Bleach at 500, 1000 and 5000 ppm. *C. difficile *spores (in ATS as an organic challenge) were used to inoculate the surface of a toilet seat (4 Log_10 _spores per site) and dried overnight. Each disinfectant was sprayed to completely wet the inoculated surface and allowed to remain in contact with the surface until completely dried (~10 minutes) with no surface wiping or allowed to remain in contact with the surface for 3 minutes followed by a manual wipe using sterile gauze that was pre-moistened with the same disinfectant. The level of residual viable spores was then assessed using the Rodac plate technique. Each experiment was performed in triplicate and each count was performed in triplicate. Results represent the mean ± standard deviation of all 9 counts. Toilet seats were inoculated with an organic challenge and spores as described in methods.

### 3. Detection of *C. difficile *spores in toilets (clinical use testing)

Each day the patient's toilet was assessed using Rodac plates containing CDMN agar (samples surface area of 25 cm^2^) for detection of the presence of *C. difficile *spores as described previously [18]. The same Rodac plate was used to sequentially sample four areas of the toilet (underside of the toilet lid, toilet seat surface and underside, as well as the inside rim of the upper portion of the toilet bowl) for a total surface are of 100 cm^2^. The identification of isolates and the confirmation of toxin production was as previously described by Alfa et al [18].

### 4. Disinfectant cleaners evaluated

Oxivir_TB_^® ^is an accelerated hydrogen peroxide (AHP) formulation that is marketed for use in healthcare, medical, industrial and institutional facilities. It has an *M. tuberculosis *label claim and is sold in a ready to use format that contains 0.5% AHP. The manufacturer recommended contact time is 1 minute. PerDiem is a stabilized hydrogen peroxide (SHP) that is used for routine housekeeping at 1:64 dilution (0.0469 final concentration of SHP at use-dilution of 1:64). There is no need for special PPE for either PerDiem or Oxivir_TB_^® ^at the use-dilutions and no wipe-off of residual disinfectant is needed. The manufacturer recommended contact time is 10 minutes for PerDiem. PerCept is a Hydrogen peroxide (HP) formulation that contains 7% HP that is to be diluted at 1:16, carries a broader spectrum of efficacy than PerDiem with a 5-minute contact time and is intended for disinfection of environmental surfaces and non-critical devices. In concentrate at 7%, this formulation has special PPE requirements; however, at the use dilution does not require special PPE. Bleach (5% household bleach containing 50,000 ppm chlorine) was also assessed at use-dilutions of 5,000 ppm, 1,000 ppm and 500 ppm (prepared fresh daily). Bleach does require special PPE at 5,000 ppm and also requires a wipe-off after appropriate contact time (no minimum manufacturer recommended contact time for household bleach).

### 5. Cleaning Compliance assessment tool

Glitterbug lotion (Brevis Inc) was used as the UV-visible marker (UVM) on the undersides of the toilet seat as an audit to ensure housekeeping staff had cleaned the toilet. The value of UVM as an audit tool of cleaning compliance has been described by Alfa et al [18]. If this marker was not removed it indicated that the toilet had not been cleaned as per the protocol therefore the culture results from the Rodac Plates taken on that day would not reflect the efficacy of the agent tested because it was not applied to the toilet. Only if the UVM was removed was the Rodac culture result included in the final data analysis.

### 6. Housekeeping protocol for cleaning toilets; routine and enhanced

PerDiem at a 1:64 use-dilution was used for routine cleaning of patient toilets. The toilet cleaning instructions required that all surfaces (including the underside of the toilet lid and seat) be sprayed to entirely wet the toilet surfaces. The solution was to be in contact with the toilet surface while the housekeeper finished cleaning the other areas of the bathroom (observational studies indicated that this required from 3 - 5 minutes). The toilet was then to be wiped using a cloth wetted with the same use-dilution PerDiem solution. The toilet was cleaned last and the cleaning cloth was not to be used for any other cleaning - it was sent for laundering. The toilet was to be cleaned once per day. The enhanced housekeeping protocol (twice daily cleaning) used by this hospital was for toilets of patients who were on isolation precautions due to CDAD. PerDiem at the standard 1:64 use-dilution was used for the enhanced protocol. The cloths used for all cleaning were cotton material of varying thickness made from old bedsheets.

### 7. Study protocol

**Figure 1:**
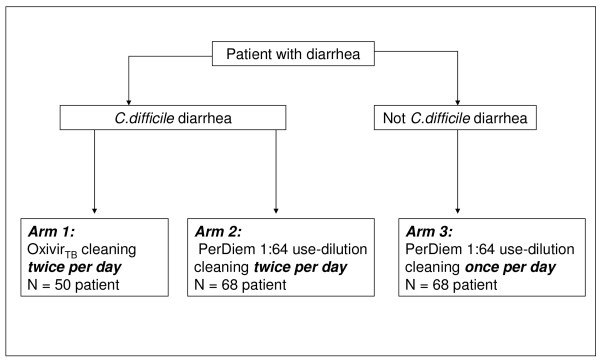
**Overview of Patient Enrolment**. All patients were followed prospectively and samples collected for five days unless the patient was discharged or died. OxivirTB^® ^has 0.5% AHP at its use-dilution, and PerDiem at a 1:64 use-dilution has 0.05% SHP.

Patients with diarrhoea were enrolled in 3 Arms as shown in Figure 1. No samples were taken from the patients and only the patient's toilet was evaluated. The Research and Ethics committee approved that informed consent of patients who used the toilet evaluated was not needed. The hospital site where this study was undertaken was a 450 bed acute care facility. All housekeeping staff were informed that a study was being done that included a UVM audit, however, they were not notified which wards or patient rooms would be assessed. It was made clear to the housekeepers that no punitive action would result from the audits performed during this study. The healthcare facility had dedicated housekeepers for each ward. Staff were instructed and signs were placed in the toilets clearly indicating that the bottles of cleaner were not be removed or changed and that only these specific bottles were to be used for the cleaning. Each day the research staff checked the rooms to ensure the proper agent was being used. The diagnostic test results for all stool samples submitted to the diagnostic lab for *C. difficile *toxin testing were reviewed on a daily basis and the toilet of each patient enrolled was prospectively evaluated for 5 days of sampling unless patient died or was discharged before the five samples were collected.

#### Data analysis

Data was entered into an excel spreadsheet and some statistical analysis determined using GraphPad Instat Program.

## Results

**Figure 2:**
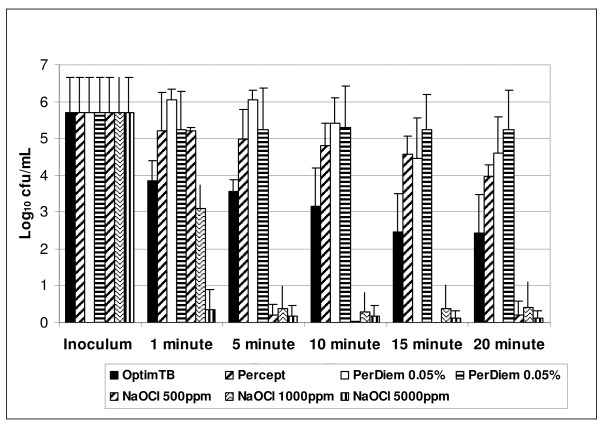
**Efficiency of chemical disinfectants on *C. difficile *spores in suspension**. The ability of Oxiv_TB _(0.5% AHP), PerCept (7% AHP), PerDiem 1:64 (0.05% SHP), PerDiem 1:16 (0.5% SHP), 500 ppm NaOCl, 1000 ppm NaOCl and 5,000 ppm NaOCl to kill *C. difficile *spores was assessed at increasing exposure times was assessed. There was no organic challenge used for this analysis. All tests were performed in triplicate and reported as the mean ± standard deviation.

**Figure 3:**
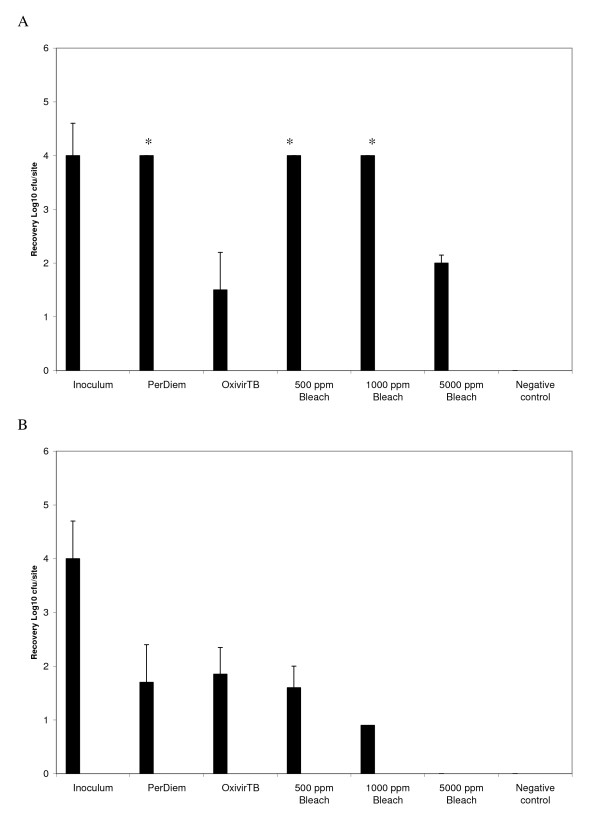
***C. difficile *spore removal from surfaces using spray only versus spray and wipe cleaning processes**. The data presented in A) represents "spray" only with no wiping of the surface prior to sampling with Rodac plates, whereas the data presented in B) represents a combination of "spraying" followed by "wiping" (single pass of the cloth) after 3 minutes of contact time. Those bars indicated by "*" represent all three samples where the number of colonies on the Rodac plate was too high to count, therefore, the samples were assumed to have ≥4log_10 _cfu/site for graphing purposes.

The objective was to select a cleaning agent that had microbial killing ability that did not require an additional rinse step after application and had some *C. difficile *sporicidal activity within a short period of time. The selected formulation would then be used for a prospective clinical study. Preliminary *in vitro *testing (Figure 2) of various disinfectant formulations using simulated-use suspension testing in the absence of an organic challenge indicated that the Oxivir_TB_^® ^formulation provided a 2-3 Log_10 _kill of *C. difficile *spores after only 1 minute exposure. The suspension killing for Oxivir_TB_^® ^was not as efficient as that achieved for bleach 5000 ppm but was equivalent to bleach 1000 ppm after 1 min. The other HP formulations tested did not have efficient spore killing ability within 1 - 5 mins. Further testing of the efficacy of *C. difficile *spore killing on surfaces in the presence of an organic challenge were also preformed (Figure 3). If a spray and wipe cleaning process was used 5000 ppm bleach was the most effective, whereas if no wiping was performed then Oxivir_TB_^® ^and 5000 ppm bleach were not statistically different in their spore killing ability. Since the objective was to identify an alternative to 5000 ppm bleach, the Oxivir_TB_^® ^formulation was selected for the intervention study as it had rapid (but not complete) sporicidal activity yet does not require special PPE and does not need the additional step of being wiped off with water (i.e. is a one step process).

The efficiency of detection of *C. difficile *spores on surfaces by Rodac plates containing CDMN agar was assessed using a known concentration of spores inoculated and dried onto plastic, tin-foil and toilet seat surfaces (Table 1).

**Table 1:** Efficacy of Rodac plates for recovering *C. difficile *spores dried onto surfaces.

	Recovery Log_10 _cfu/site (± std)*	% recovery (+ std)
1. Plastic Petri dish	2.041 (0.125)	80.81 (4.96)

2. Tin foil	2.186 (0.015)	86.6 (0.61)

3. Toilet seat	2.162 (0.008)	85.7 (3.03)

* The surface area inoculated was equivalent to the surface area sampled when using the Rodac plate. All experiments were performed in triplicate. The average inoculum per surface area inoculated (i.e per site) was Log_10 _2.524 ± 0.009 cfu/site. A range of surface inoculation levels were evaluated, but higher inoculum (e.g. ≥ 10^3 ^cfu/site) of *C. difficile *spores produced growth that was too numerous to count when sampled using Rodac plates.

The ward intervention study (Figure 1) was undertaken between December 2005 and July 2007. Patients in Arms 1, and 2 were prospectively followed while on isolation precautions for at least 1 week post diagnosis of CDAD unless discharged earlier or taken off of isolation precautions. Patients in Arm 3 (not on isolation) were prospectively followed for at least 1 week unless discharged earlier. Although patients who are treated for CDAD are known to shed spores for prolonged periods, the expectation was that the time of highest likelihood of having *C. difficile *spores shed into the toilet was during the first week post-diagnosis.

**Figure 4:**
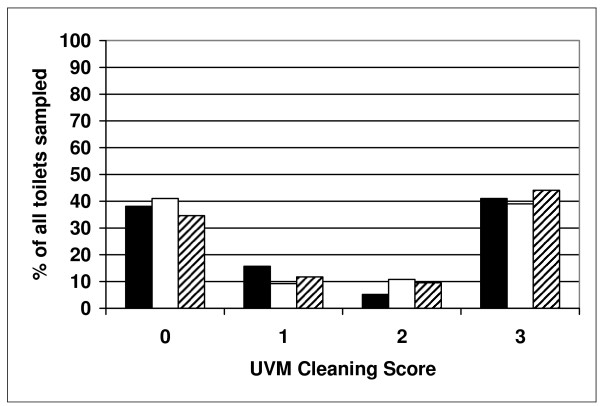
**Overall UVM audit to assess the cleaning efficacy in each arm of the study**. The data represents the overall average percent of samples in each Arm showing 0, 1, 2 or 3 as the UVM cleaning score. This is based on data from the first 5 days of samples for each patient in each Arm. This represents 133 samples from 50 patients in Arm 1 (solid bars), 254 samples from 68 patients in Arm 2 (white bars) and 179 samples from 68 patients in Arm 3 (hatched bars).

**Figure 5:**
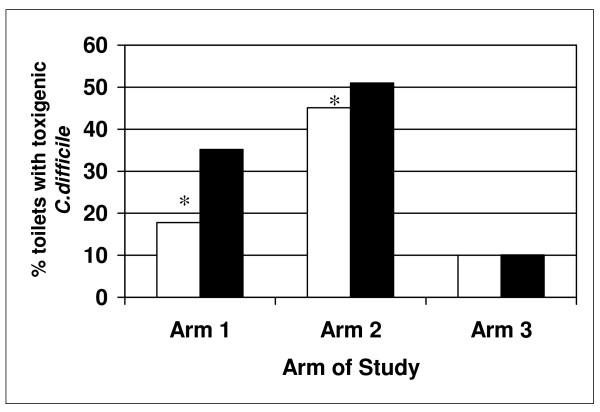
**Effect of cleaning protocol on *C. difficile *detection in toilets**. Data for toilets where UVM was 3 (no cleaning) was compared to toilets that received some cleaning (UVM of 0, 1, 2). See Materials and Methods and Figure 1 for differences in patient population and cleaning protocols used for Arms 1 to 3. Arm 1 had 50 patients enrolled and a total of 133 samples (79 samples with UVM <3 and 50 samples with UVM = 3), Arm 2 had 68 patients and 254 samples (153 samples with UVM <3 and 101 samples with UVM = 3) and Arm 3 had 68 patients enrolled with 179 samples (100 samples with UVM <3 and 79 with UVM = 3. Arm 1 showed significantly lower detection of toxigenic *C. difficile *detected compared to Arm 2* (p = 0.0023, CI of 0.4589 to 0.8482). The white bars represent results for samples with a UVM <3 (some cleaning) and the solid bars represent results from samples with a UVM = 3 (no cleaning).

To ensure the cleaning agents (both AHP and SHP) were applied to the toilets, a UV visible marker (UVM) was used on the underside of the toilet seat following the protocol of Alfa et al [18]. Our data (Figure 4) showed 30 to 41% of the toilets had a UVM score of 3 indicating the marker was not removed, therefore, not cleaned according to the housekeeping protocol. The overall average UVM scores were 1.48, 1.40, 1.48 and 1.63 for Arms 1, 2, and 3 respectively (not significantly different by ANOVA, p = 0.4253). To ensure reliable conclusions, the data on *C. difficile *culture results was stratified based on UVM audits. Figure 5 summarizes the data for *C. difficile *detection in Arms 1, 2, and 3 for samples collected from toilets where the UVM confirmed there had been some level of cleaning (i.e. UVM <3) versus when the UVM was 3 (no cleaning). Arm 1 (intervention agent) had significantly lower spore levels compared to Arm 2 (currently used protocol).

## Discussion

Our study is the first to incorporate UVM as an audit to confirm that cleaning of the toilet was done during the assessment of an alternative cleaning formulation. Our data indicated that the compliance with the housekeeping cleaning policy (i.e. UVM score <3) was about 60% which is consistent with what has been previously reported in regular as well as ICU rooms [19-21]. Despite the policy requiring cleaning of toilets twice per day for patients with CDAD, this was not being achieved 40% of the time. Recently a number of published reports [18,22-24] have indicated that the currently accepted benchmark of "visibly clean" is an inadequate audit approach in healthcare facilities. Alternative audit tools suggested include ATP bioluminescence and culture [22,23] with cleaning considered inadequate at ATP levels ≥ 500 RLU/cm^2 ^or when culture showed >1 cfu/cm^2 ^of *C. difficile. *Although using ATP based audit tools [22-24], or culture may provide a more direct measure of the residual bioburden, the UVM method provides an inexpensive and easy method for sites to accurately audit cleaning compliance (i.e. physical wiping of the surface) and this assessment is critical when comparing a possible alternative disinfecting/cleaning agent to a current agent.

Although Rodac plates containing CDMN provide excellent recovery of *C. difficile *spores from toilet surfaces (86%), it should be noted that *C. difficile *spores in a patient-used toilet will not be evenly distributed and our Rodac sampling method (sampled 100 cm^2^) represented only about 10% of the total toilet surface area that could be contaminated. Dispite this caveat the Rodac sampling method provides a good indication of the level of spore contamination as it samples the sites most likely to be contaminated after patient-use or after the contents of a bedpan are disposed of in the toilet.

The daily monitoring of *C. difficile *spores in the patient toilet using Rodac plates provided direct assessment of the impact of the cleaning agent on spore removal/killing. Physical cleaning is not sufficient alone to eliminate spores and use of a disinfectant may be needed to provide an efficient means of reducing the level of environmental spores. Although chlorine releasing agents are optimal for spore killing even 5,000 ppm bleach has been reported as not totally effective [1,7,9,23]. The previous published clinical studies did not have audits to ensure cleaning compliance, as such the suboptimal results obtained using 5,000 ppm bleach (up to ~20% still having *C. difficile*) may be due to lack of housekeeping compliance. However, [9] Eckstein reported that even when research staff performed the cleaning that 5000 ppm bleach was not totally effective at eradicating *C. difficile *spores from patient toilets (10% still having *C. difficile*). Our data indicated that although bleach at 500 to 5,000 ppm was effective at killing *C. difficile *spores in suspension in 5 minutes, it was not as effective when surface testing in the presence of an organic challenge was used. Perez et al [13] have shown that 5,000 ppm bleach needs 5 - 8 mins to provide a 6 Log_10 _reduction in *C. difficile *spores. Oxivir_TB_^® ^was as effective as 5000 ppm bleach when tested on surfaces with an organic challenge without physical wiping (neither was completely effective without wiping). Perez et al's [13] data also support the value of AHP for *C. difficile *spore killing. However, the formulation they tested is designed for medical device reprocessing and therefore requires special PPE as well as a second wipe with water to remove the AHP residuals. Neither the AHP formulation (Oxivir_TB_) or the SHP formulation (PerDiem) used for the current study require special PPE or a second wipe to remove residuals. There have been published reports that the use of AHP disinfecting cleaners have been associated with outbreak control and reduced rates of CDAD [11].

Our study is the first to provide data that a one stage cleaning process using an AHP (0.5%) formulation can significantly reduce the load of *C. difficile *spores in the toilets of patients with CDAD during non-outbreak conditions. Our study demonstrated that the AHP intervention resulted in toilet spore levels (27.8%) nearly equivalent to those found when Eckstein et al [9] used 5,000 ppm bleach (~20%). It is critical that for "intervention studies" that cleaning compliance is audited. If the toilet has not been cleaned, results should not be included in the analysis of the intervention as the conclusions may be misleading.

Panessa [25] et al have reported that despite not being metabolically active, spores do attach to surfaces especially when sporulation is initiated. This ability of spores to attach combined with lack of cleaning compliance by housekeeping staff may be the basis for accumulation of spores and high positivity rates in toilets and high-touch areas of the rooms of patients with CDAD [1,7,9,11,26]. Despite a housekeeping policy requiring enhanced frequency of cleaning (twice per day) for CDAD patient isolation rooms, *C. difficile *spores were still detected in approximately 50% of toilets. Although this may be partly due to the housekeeping protocol not stipulating 10 minutes contact time with the use-dilution of PerDiem (as per manufacturer's contact time recommendations) it is unlikely that re-wetting the surface to provide 10 minutes contact time would have improved elimination of *C. difficile *spores as our preliminary testing showed that this formulation had essentially no ability to kill *C. difficile *spores even after 20 minutes exposure in suspension testing. The lack of ability of PerDiem (routinely used throughout the hospital) to eliminate spores may also explain why the background level of *C. difficile *is 10% even in rooms of patients without CDAD. Alternatively, this may be the lowest environmental level of *C. difficile *spores that can be achieved in healthcare facilities as our background level of 10% was similar to the residual level of spores when research staff did toilet cleaning in patient rooms using 5,000 ppm bleach [9]. The efficacy of any cleaning/disinfecting agent tested is dependent on physical action. We recommend that healthcare facilities need to ensure that adequate time is allowed for cleaning of patient rooms and that adequate audits are used to ensure compliance with the cleaning protocol.

## Conclusion

In conclusion, our data demonstrated that the use of an agent with some sporicidal activity for cleaning resulted in significantly reduced *C. difficle *spore levels in toilets of patients with CDAD during non-outbreak conditions. Infection transmission within healthcare will remain problematic if the role of housekeeping remains undervalued and if they are not provided with adequate audit tools such as UVM to ensure sustained cleaning compliance.

## Competing interests

The ECR and all funds for this study were provided by Advanced Sterilization Products, a Division of Ethicon, a Johnson and Johnson company. MJA has undertaken contract research projects (unrelated to the current publication) for a variety of companies including; 3 M, bioMerieux, Olympus, Case Medical, Intelligent Hospital Systems, Johnson & Johnson, Novaflux, STERIS and Virox. No monies from the current or past research contracts have gone to MJA, they were administered through the St. Boniface Research Centre and were used for research related expenses only. MJA has been a sponsored conference speaker for numerous conferences and she has acted as a paid consultant for preparation of a one time literature review for STERIS in 2003 and for providing a one time educational microbiology workshop for 3 M staff in 2006.

The other authors have not acted as consultants and have no financial or other link with any company.

## Authors' contributions

MA conceived the protocol and was involved in data analysis and had a primary role in manuscript writing. EL & GH were involved in data analysis and manuscript writing. AW, CD & PD were involved in ward sampling, data analysis and manuscript proofing. All authors have read and approved the final manuscript.

## Pre-publication history

The pre-publication history for this paper can be accessed here:

http://www.biomedcentral.com/1471-2334/10/268/prepub

## References

[B1] McFarland LV Beneda HW Clarridge JE Raugi GJ Implications of the changing face of Clostridium difficile disease for health care practitioners American journal of infection control 2007 35 4 237 253 10.1016/j.ajic.2006.06.004 17482995

[B2] Miller MA Hyland M Ofner-Agostini M Gourdeau M Ishak M Morbidity, mortality, and healthcare burden of nosocomial Clostridium difficile-associated diarrhea in Canadian hospitals Infect Control Hosp Epidemiol 2002 23 3 137 140 10.1086/502023 11918118

[B3] Dallal RM Harbrecht BG Boujoukas AJ Sirio CA Farkas LM Lee KK Simmons RL Fulminant Clostridium difficile: an underappreciated and increasing cause of death and complications Annals of surgery 2002 235 3 363 372 10.1097/00000658-200203000-00008 11882758 PMC1422442

[B4] Severe Clostridium difficile-associated disease in populations previously at low risk--four states, 2005 Mmwr 2005 54 47 1201 1205 16319813

[B5] Loo VG Poirier L Miller MA Oughton M Libman MD Michaud S Bourgault AM Nguyen T Frenette C Kelly M A predominantly clonal multi-institutional outbreak of Clostridium difficile-associated diarrhea with high morbidity and mortality The New England journal of medicine 2005 353 23 2442 2449 10.1056/NEJMoa051639 16322602

[B6] (CPL) MPHCCPL Communicable Disease Report, 2006 2006

[B7] Dubberke ER Reske KA Noble-Wang J Thompson A Killgore G Mayfield J Camins B Woeltje K McDonald JR McDonald LC Prevalence of Clostridium difficile environmental contamination and strain variability in multiple health care facilities American journal of infection control 2007 35 5 315 318 10.1016/j.ajic.2006.12.006 17577478

[B8] Sunenshine RH McDonald LC Clostridium difficile-associated disease: new challenges from an established pathogen Cleveland Clinic journal of medicine 2006 73 2 187 197 10.3949/ccjm.73.2.187 16478043

[B9] Eckstein BC Adams DA Eckstein EC Rao A Sethi AK Yadavalli GK Donskey CJ Reduction of Clostridium Difficile and vancomycin-resistant Enterococcus contamination of environmental surfaces after an intervention to improve cleaning methods BMC infectious diseases 2007 7 61 10.1186/1471-2334-7-61 17584935 PMC1906786

[B10] Whitaker J Brown BS Vidal S Calcaterra M Designing a protocol that eliminates Clostridium difficile: a collaborative venture American journal of infection control 2007 35 5 310 314 10.1016/j.ajic.2006.08.010 17577477

[B11] Boyce JM Environmental contamination makes an important contribution to hospital infection The Journal of hospital infection 2007 65 Suppl 2 50 54 10.1016/S0195-6701(07)60015-2 17540242

[B12] Fawley WN Underwood S Freeman J Baines SD Saxton K Stephenson K Owens RC Jr Wilcox MH Efficacy of hospital cleaning agents and germicides against epidemic Clostridium difficile strains Infect Control Hosp Epidemiol 2007 28 8 920 925 10.1086/519201 17620238

[B13] Perez J Springthorpe VS Sattar SA Activity of selected oxidizing microbicides against the spores of Clostridium difficile: relevance to environmental control American journal of infection control 2005 33 6 320 325 10.1016/j.ajic.2005.04.240 16061137

[B14] (PHAC) HC Hand Washing, Cleaning, Disinfection and Sterilization in Health Care 1998 24S8 11195271

[B15] (PIDAC) PIDAC Best Practices for Cleaning, Disinfection and Sterilization in All Health Care Settings 2006

[B16] Wilcox MH Fawley WN Wigglesworth N Parnell P Verity P Freeman J Comparison of the effect of detergent versus hypochlorite cleaning on environmental contamination and incidence of Clostridium difficile infection The Journal of hospital infection 2003 54 2 109 114 10.1016/S0195-6701(02)00400-0 12818583

[B17] Alfa MJ Olson N Buelow-Smith L Simulated-use testing of bedpan and urinal washer disinfectors: evaluation of Clostridium difficile spore survival and cleaning efficacy American journal of infection control 2008 36 1 5 11 10.1016/j.ajic.2007.04.277 18241730

[B18] Alfa MJ Dueck C Olson N Degagne P Papetti S Wald A Lo E Harding G UV-visible marker confirms that environmental persistence of Clostridium difficile spores in toilets of patients with C. difficile-associated diarrhea is associated with lack of compliance with cleaning protocol.e BMC infectious diseases 2008 8 64 10.1186/1471-2334-8-64 18474086 PMC2390558

[B19] Carling PC Briggs J Hylander D Perkins J An evaluation of patient area cleaning in 3 hospitals using a novel targeting methodology American journal of infection control 2006 34 8 513 519 10.1016/j.ajic.2005.09.001 17015157

[B20] Carling PC Parry MF Von Beheren SM Identifying opportunities to enhance environmental cleaning in 23 acute care hospitals Infect Control Hosp Epidemiol 2008 29 1 1 7 10.1086/524329 18171180

[B21] Carling PC Von Beheren S Kim P Woods C Intensive care unit environmental cleaning: an evaluation in sixteen hospitals using a novel assessment tool The Journal of hospital infection 2008 68 1 39 44 10.1016/j.jhin.2007.09.015 18069083

[B22] Dancer SJ Importance of the environment in meticillin-resistant Staphylococcus aureus acquisition: the case for hospital cleaning The Lancet infectious diseases 2008 8 2 101 113 10.1016/S1473-3099(07)70241-4 17974481

[B23] Cooper RA Griffith CJ Malik RE Obee P Looker N Monitoring the effectiveness of cleaning in four British hospitals American journal of infection control 2007 35 5 338 341 10.1016/j.ajic.2006.07.015 17577482

[B24] Griffith CJ Obee P Cooper RA Burton NF Lewis M The effectiveness of existing and modified cleaning regimens in a Welsh hospital The Journal of hospital infection 2007 66 4 352 359 10.1016/j.jhin.2007.05.016 17655976

[B25] Panessa-Warren BJ Tortora GT Warren JB High resolution FESEM and TEM reveal bacterial spore attachment Microsc Microanal 2007 13 4 251 266 10.1017/S1431927607070651 17637074

[B26] Dettenkofer M Spencer RC Importance of environmental decontamination--a critical view The Journal of hospital infection 2007 65 Suppl 2 55 57 10.1016/S0195-6701(07)60016-4 17540243

